# Study on influence factors of public participation willingness in substation project based on integrated TPB-NAM model

**DOI:** 10.3389/fpsyg.2022.999229

**Published:** 2022-11-09

**Authors:** Xin Ma, Junpeng Li, Fuli Guo, Caocao Cui, Tengfei Chen, Fan Xv, Wenbin Wang

**Affiliations:** ^1^School of Management and Economics, North China University of Water Resources and Electric Power, Zhengzhou, China; ^2^Business School, Central South University, Changsha, China; ^3^Resource System Optimization and Decision Research Center, North China University of Water Resources and Electric Power, Zhengzhou, China

**Keywords:** substation, NIMBY, TPB, norm activation model, integrated model

## Abstract

Public infrastructure, such as substations, is crucial for the advancement of the economy and society. However, the “not in my backyard” phenomenon is causing concern among the population, and these two things are at odds with one another. This study aims to investigate the driving mechanism that influences participation willingness of the public in order to promote the construction of substations, so the study proposes an integration model based on the planned behavior theory and the normative activation theory. Moreover, a structural equation model is created using the two dimensions, namely, social altruism and personal egoism, while data of 568 questionnaires are used for empirical research in combination with the “Decision-Making Trial and Evaluation Laboratory” method; these data are collected in the surrounding areas of three 110kV substations in Jiaozuo city, China. The key factors that affect participation willingness of the public are discussed, and the study demonstrates that the model is most significantly impacted by public trust, which is an *a priori* variable. Furthermore, the direct path coefficient of personal norms on participation willingness is the largest, which confirms that increased moral responsibility has a beneficial effect on project execution, and subjective norms contribute to the improvement of the assessment model overall since they are the main variables with the largest centrality degree in the system. The findings of this research better our understandings about the mechanism of “not in my backyard” and offer practical implications for its dissolution. On the basis of this, we present pertinent policy proposals for the “not in my backyard” effect that develops during the construction of public infrastructure.

## Introduction

A substation is the key component of the power system, which undertakes the tasks of receiving and distributing electric power and converting voltage levels. It plays an important role in the rapid development process of urbanization and industrialization in China. In terms of technical requirements, the substation should meet the load it supplies and allow for a certain amount of reserve capacity. It is also important to note that the loads supplied should be within the permissible supply radius. The larger the urban expansion and the faster the economic development, the more substations need to be built. Only under these conditions, the electrical power industry can meet the increasing load demands of the city, while optimizing the structure of the electrical power grid and ensuring its stable operation. However, perception of risks of the public arising from substations is not consistent with the objective risk due to the limitations of their knowledge structure in relation to electromagnetic radiation (Liu and Tang, 2020). Driven by online public opinions and complex information, this prejudice will continue to be amplified and may lead to public concern, public opposition, and resistance, and even social conflict once the public perceives that the living environment, personal health, and safety are compromised (Yu et al., 2022). This phenomenon is called “not in my backyard” (NIMBY), which usually refers to a project that has negative externalities and the residents in the vicinity of the project are trying to protect themselves, thus creating resistance and resisting behavior toward the project (Li and Li, 2021). There is a conflict between the NIMBY effect of the substations and the desire of the public for environmental quality, and this not only delays economic and social development but also leads to a decline in the public trust on the government. Moreover, this conflict can affect the construction of subsequent projects and further expand the NIMBY effect. Therefore, this study explores the factors affecting willingness of the public to participate in substation projects from the perspective of their trust, and analyzes effective ways to address NIMBY, and the purpose of this article is to provide a reference for solving the problem of social failure arising in public infrastructure construction.

This study is innovative in the following aspects: First, we examine the behavior and willingness of the public to participate in substation projects from the behavioristic psychology perspective. This study reveals the mechanisms by which different factors influence willingness of the public to participate in substation projects in the context of public trust in China. Second, this study echoes the view of Kopaei et al. (2021) that “the combination of normative activation models and theory of planned behavior models can more accurately predict public behavior with respect to NIMBY projects.” In this context, we construct an integrated TPB-NAM model, empirically test the model fit and applicability, and enrich the research on the NIMBY effect. Third, the traditional Dematel method mainly uses expert scoring to construct the direct correlation matrix, but the scoring process is inevitably influenced by subjective attitude. Therefore, the study agrees with the idea that “there is non-objectivity in assessing the correlation coefficient between influence factors through expert scoring, which limits the application of Dematel method” (Song et al., 2020). This study combines the structural equations with the Dematel method, which not only inherit the advantages of the structural equations in dealing with the influence factors between complex latent variables but also circumvent the shortcomings of the non-objective property of the Dematel method, expand the depth of analysis using structural equations, and also internalize the logical relationship between the variables, which can effectively distinguish the difference between cause factors and result factors and judge the importance of each influence factor. This study is organized into six sections. In the first section, we discuss the context of the study. The second section describes literature review, and the next section describes the methods. After that, results are demonstrated, followed by discussion. Conclusion ends the whole article.

## Literature review

### Characteristics of NIMBY

The construction of NIMBY projects has a significant positive impact on the society as a whole, while the residents around the NIMBY projects bear a significant risk, which leads to an imbalance in the distribution of benefits across geographies and communities. It is a zero-sum game in which the interests of one side are enhanced, while the interests of the other side are compromised (Zhao et al., 2017). According to the theory of “politics of scale,” the essence of the NIMBY effect is the process of scale resistance of different interested parties (Wang et al., 2017). NIMBY projects can have an impact on the public in terms of residential amenities, visual impact, and noise and are often rejected by nearby residents (Wang et al., 2021). From the public point of view, the main reason why people avoid the NIMBY projects is the sense of relative deprivation of basic benefits (Zhao et al., 2021). In recent years, in China, the public has become increasingly aware of their rights and self-protection and is increasingly opposed to the NIMBY projects, and the NIMBY effect has become an important social governance issue (He and Lin, 2019). First, conflicts over the projects with NIMBY are becoming more frequent. Second, there are more types of NIMBY projects causing conflicts, such as substations, garbage disposal center, and hospitals. Third, there are diverse public demands on the problems caused by the NIMBY effect. Fourth, the intensity of conflicts is escalating (Zheng et al., 2015). Since governments are rational political homo economicus, they always play a multi-party game around “policy risk–reward” in controlling policy resistance (Yan and Chen, 2016). Faced with the balance between “policy objectives” and “social stability,” governments must consider not only whether the operation of NIMBY projects can contribute to the achievement of public objectives but also how to deal with conflict, as well as effective responsiveness and binding response strategies (Wan et al., 2020).

### NIMBY-related factors

The causes of conflicts arising from the NIMBY projects are not simply a superposition of resources, interests, and information but a complex dynamic process of risk aggregation to outbreak, covering a wide range of individual characteristics, psychological perceptions, information dissemination, social interaction, etc. (Li and Li, 2021). First, individual characteristics and psychological states become intrinsic driving forces in the formation of the NIMBY effect. For example, environmental beliefs, media catalysts, and trust in government all become influential factors in accepting or opposing the NIMBY projects (Wang and Ye, 2020). Mclaughlin and Cutts (2018) identified health risks, property risks, perceived risks, quality of life risks, and perceived frustrations as factors that contribute to the public's negative identification with the NIMBY effect. According to the hierarchical dependency expected utility theory, negative emotions such as pessimism and anxiety increase the fear of uncertainty, which leads to the outbreak of conflict due to the NIMBY effect (Li and Liang, 2018). Second, perceived benefits are the convenience and wellbeing that the public infrastructure brings to society as a whole. Its positive benefits are an effective way to increase willingness of the public to accept it. The projects bring risks as well as benefits to the public, such as increased employment and improved local infrastructure. The attitude of the public toward NIMBY projects is influenced not only by the perceived risks but also by the perceived benefits (Cowan, 2010). Public attitudes toward these projects often depend on their judgments and trade-offs between the perceived risks and benefits (Simsek et al., 2014). Third, the external environment also provides an explanatory perspective for the NIMBY effect. For example, information exchange and knowledge reinforcement are important antecedents for weakening the negative effects of NIMBY, and information and knowledge show an inverted U-shaped relationship with public perceptions of risk induced by the NIMBY effect (Liu et al., 2021); that is, risk perceptions are lower among those who “know nothing” about the risks of the NIMBY effect or among professionals who “know everything” about the risks, while people who know something about the risks feel more stressed by the NIMBY effect and are more likely to resist it (Holleran, 2021). In addition, the decision-making process of the government is more focused on scientific and technological requirements but lacks the supporting facilities to maintain environmental justice, which is an important cause of public acceptance (Liu B. et al., 2018).

### Preventive measures for NIMBY

The choice of policy instruments is the result of a combination of the planning capacity of the country and the policy subsystem, where the government takes a series of measures to implement policies and projects, and these measures may have an impact beyond the policy decision itself and have a direct impact on the successful implementation of the project (Lu et al., 2019). The response of the government to the NMBIY effect consists of three types of measures: mandatory, progressive, and concessions (Zhang and Tong, 2014). First, in the “mandatory” measure, the government enforces the construction of the NIMBY projects. In this case, the government has a monopoly on decision-making and selectively uses information strategies, such as promoting benefits and avoiding risks, the likelihood of public participation is low, and negative sentiment is strong and intense, which means that the government is vulnerable to a reactive situation (Ru, 2020). Second, in the “progressive” measure is a more moderate approach that takes a step-by-step effort to advance the construction of the NIMBY projects, for example, formulation of a reasonable compensation policy by the government (Rouhani et al., 2022). Third, in the “concessions” measure, the government does nothing in response to public resistance to NIMBY projects, which will undoubtedly result in loss of government investment.

### TPB and NAM

The TPB is developed on the basis of the reasoned action theory (RAT). The TPB has been widely used in various studies related to personal behavior (Lee et al., 2021; Wan et al., 2022). It is often used to explain the main factors that influence personal behavior in the decision-making process (Icek, 2011). According to this theory, one's behavior is determined by one's own willingness to perform or achieve a particular behavior, that is, behavioral intention (Thompson et al., 2020). It is influenced by three independent factors: attitudes to behavior (ATB), subjective norms (SNs), and perceived behavioral control (PBC) (Wu and Chen, 2005). ATB is one's pre-evaluation of the outcome of behavior, that is, a person's prior judgment of the pros and cons of behavior in terms of personal preference (Cheng et al., 2020). SN is defined as pressure from others (usually close family and friends) or group expectations on one's behavioral decision-making process to perform or not to perform it (Liao et al., 2007). PBC refers to the evaluation of one's internal control, expressed as an actor's assessment of the ease of implementing the current decision based on past experience (Lim et al., 2020). Zhang et al. (2020) used the TPB to study the formation mechanism of residents' participation behavior toward NIMBY projects. Following an empirical study using the TPB, Yancy (2018) argued that public attitudes toward NIMBY projects are actually a reflection of the acceptability of risk; the attitudes are both the psychological basis of the NIMBY effect and the psychological reflection of person toward particular objects.

NAM has been used to effectively explain pro-social and altruistic behavior (Jabilles et al., 2019). Regarding NAM, the altruistic behavior is the internalization of underlying social norms in people's lives as a collection of social responsibility, moral obligation and values, etc. (Rosenthal and Yu, 2022). Although society encourages helpful behavior, it does not mean that all people can follow society's dictates to achieve effective practice; therefore, a person's ability to display altruistic behavior is individually heterogeneous and depends on the positive influence of personal norms (PNs), which will ultimately be reflected in the intention (INT) (Hao and Yang, 2018). The activation of personal norms, which are both moral obligations and internalized social norms, depends on two core variables: awareness of consequences (AC) and attribution of responsibility (AR). AC refers to the extent to which a person expects that the behavior in which he or she is about to engage may have positive or negative effects, and AR refers to a person's sense of responsibility for the consequences of undesirable behavior (Kokolakis, 2017). NAM is widely used to study various pro-environmental behaviors of the public, for example, research into issues such as energy saving, transport mode choice, and waste recycling (Khan et al., 2019).

Despite these valuable results, most of the literature examining the NIMBY effect focuses on public attitudes and then discusses public intentions in terms of mediated intentions, subjective norms, and perceived behavioral control, but rarely considering public altruistic behavior. Whereas NAM considers altruism to precede egoism, TPB emphasizes personal utility; there are differences between the two models, as well as different emphases in their application. If the two models are combined, researchers can explore factors that influence public behavior from multiple perspectives: First, what are the key factors that influence public acceptance of a substation project? Second, can public trust play an important role in substation projects with public participation? Third, does the integrated TPB-NAM model provide a solid theoretical basis and explanatory validity for measuring public acceptance of NIMBY projects such as substations? Finally, can this article provide effective recommendations for policymakers to deal with social conflicts and communication dilemmas in urban development?

## Methods

### Integrated TPB-NAM model

The constraints on the objective environment and the impact of personal norms are rarely taken into consideration because the TPB emphasizes the influence of egoism but ignores the crucial role of irrational and altruistic motivation in behavior shaping, and the effectiveness of the explanation in its application to pro-social behavior is still up for debate. In contrast to the TPB, which has a limited range of explanations, NAM is a classical theoretical framework for explaining pro-social altruistic behavior. Therefore, researchers suggest combining the TPB and NAM to strengthen the explanatory effectiveness for one's intention to engage in pro-environmental action (Shen et al., 2020). The explanatory effectiveness of the initial model is greatly increased if the personal norms variable is included in the TPB model (Yazdanpanah and Forouzani, 2015). Furthermore, explanatory effectiveness is also improved when moral obligation is introduced into the model (Kim et al., 2018).

**Figure 1:**
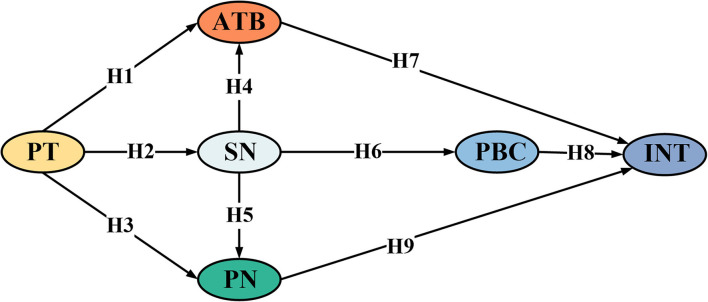
Theoretical hypothesis model.

The findings are not entirely accurate when behavior of the public is simply examined from the perspective of egoism or altruism (Qin et al., 2020). As a result, if the TPB and NAM are combined, one's propensity to engage in social activity may be predicted and explained more effectively (Wang et al., 2018). The attribution of responsibility is typically viewed as a modulator between personal norms and actions and is seldom incorporated into the integrated framework of the TPB and NAM (Li and Wu, 2019), so it is not discussed in the integrated model in this article. Why is the awareness of consequences not included? First of all, it focuses on representing the expected degree of positive or negative influence on one's behavior to participate, and there is some functional overlap with the attitude variables in the integration framework. Second, the key issue of this study is to discuss the cognitive bias of the public and the deficiency caused by the lack of knowledge about substation construction. This research designs a TPB-NAM integration model including public trust (PT) variables to study the primary factors influencing willingness of the public to participate in the substation project from both egoism and altruism aspects. The research model of this study is given in Figure 1.

### Hypothesis

Trust is often defined as a relationship of recognition that relied on relevant institutions or people who have the power to make decisions and adopt technical and policy instruments to implement them (Aracil et al., 2018). In general, people lack expertise in the construction of public infrastructure such as substations (Yao et al., 2021), and the public trust in authorities and government departments will influence their pre-evaluation of the project and their willingness to participate in the NIMBY projects (Eguchi, 2020). An increase in the level of public trust may contribute to a positive social evaluation of the implementation of substation projects, as well as positively influencing ones' willingness to participate and further stimulating their sense of moral responsibility (Stehlik, 2009; Cheng and Zhang, 2021). Therefore, the following hypotheses are proposed:

H1: Public trust has a positive influence on ATB.
H2: Public trust has a positive influence on SN.
H3: Public trust has a positive influence on PN.

Subjective norm is the antecedent variable of PN and has a direct role in verifying that one's decision-making behavior is consistent with one's social value, and it is usually used as a measure of whether social behavior is consistent with right morals and values (Bamberg et al., 2007; Zhang et al., 2018). The relationship between SN and ATB shows that the perceptions of others or groups may affect one's ATB (Rezaei et al., 2019). If friends and family share the view that substations can improve the stability of electricity consumption and promote economic and social development, then due to herd mentality and dependency, an individual may hold the same attitude toward substation projects. The direct link between SN and PBC exhibits that the endorsement of substation projects by others, in response to society, institutional, and other pressures, may reduce the perceived barrier encountered by one's decision-making process (O'Neil, 2021). Based on this, the following hypotheses are proposed:

H4: SN has a positive influence on ATB.
H5: SN has a positive influence on PN.
H6: SN has a positive influence on PBC.

One's positive attitude toward substation projects would increase one's willingness to participate (Komendantova and Battaglini, 2016; Fu et al., 2022). One's behavioral decision is always affected by one's own perception (Zhang et al., 2017). When people have the right and more knowledgeable structure, that is, they are aware of the need for substation construction, and the public has a responsibility and obligation to support the development of the city, which will strengthen their willingness to participate (Li and Liang, 2018). Furthermore, people are more likely to be willing to participate when they feel pressure from the society that current electrical load needs to be satisfied by building substations and that public support for the substation projects will contribute to social progress. On the basis of the previous discussion, the following hypotheses are proposed:

H7: ATB has a positive influence on INT.
H8: PBC has a positive influence on INT.
H9: The PN has a positive influence on INT.

### Data collections and sample

110kV substations are the most common terminal substations in the urban grid structure and play an important role in ensuring the reliability of power supply and power quality. In this work, three 110kV substation projects in Jiaozuo city, China, are selected for study, namely, GL substation, GC substation, and LZ substation, where a large number of supporting facilities such as schools, residential areas, and supermarkets exist within a 3-km radius. These substations were built to solve the difficulties caused by the increased electrical power load of businesses and residents and were in full operation in 2020. A total of six survey teams, each consisting of two people, are organized to conduct a week-long face-to-face random survey in areas with high population around the substation project areas, and 568 valid questionnaires are obtained after collating and eliminating uncritical questionnaires.

In order to minimize possible response bias, the instruction that “there are no right or wrong answers; only your personal opinions matter” was emphasized in the cover letter. On completing the questionnaire anonymously, voluntary participants were assured that all individual responses would be kept confidential. Table 1 summarizes the demographic characteristics of the overall sample participating in this survey.

**Table 1:** Sample profiles of the respondents.

**Category**	**Classification**	**Frequency**	**%**
Gender	Male	274	48.20%
	Female	294	51.80%
Age	<23	83	14.60%
	Between 23–35	237	41.70%
	Between 36–55	199	35.00%
	More than 55	49	8.60%
Education	Below middle school	92	16.20%
	High school	228	40.10%
	Graduate	166	29.20%
	MS/MPhil or PhD	82	14.40%
Whether you own a property	Yes	274	48.20%
or intend to buy one in the near future?	No	294	51.80%
Have you understood the role	Yes	173	30.50%
of the substation beforehand?	No	395	69.50%
Have you ever been involved	Yes	34	6.00%
in the construction of a substation	No	534	94.00%
Is there a substation near	Yes	181	31.90%
where you live?	No	132	23.20%
	No information	255	44.90%

The percentages of male and female subjects are almost equal (48.20 and 51.80%, respectively). A majority of them were in the age range of 23~35 and 36~55 years (41.70 and 35.00%, respectively). Across the sample, most people had high school education (40.10%), a graduate degree (29.20%), or MS/MPhil or PhD (14.40%). In this sample, 48.2% of the respondents had purchased or planned to purchase a property, but 69.50% of the respondents said they only knew that the substation was used to supply electricity and were unaware of the specific role and technical requirements of the substation, indicating a continued lack of public understanding of public infrastructure projects. This sample comprise respondents with a wide variety of sociodemographic backgrounds. More specifically, it is believed that the raw survey data collected in this empirical study can carry theoretical and practical implications for this type of research.

### Measures

The measurement scales and indicators adopted for the present study with the goal of measuring all the studied construct variables are validated by previous studies. Following Liu Y. et al. (2018), a four-item PT measurement scale is used, and the respondents are asked to express their degree of agreement to site selection process/equipment and technology/expert analysis/construction process. A three-item ATB measurement scale using in this study mainly refers to the research views of Zhang et al. (2017), Rezaei et al. (2019), and Qin et al. (2020), in order to ascertain whether one would approve or disapprove substation is valuable and useful for living quality or social development. Based on Rezaei et al. (2019) and Qin et al. (2020)'s study, a three-item SN measurement scale is used to measure the social pressure felt by one person whether to support the construction of the substation. Following Zhang et al. (2017) and Qin et al. (2020), a three-item PN measurement scale is also used to measure one's own judgment on the construction of a substation. Adapted from Zhang et al. (2017), Rezaei et al. (2019), and Qin et al. (2020)'s study, a four-item PBC measurement scale is used to measure one's belief in the inherent difficulty in completing a certain behavior, meaning that a person makes a decision to participate/not participate in the construction of a substation not simply under his/her volitional control. Following Qin et al. (2020), a three-item INT measurement scale is used to find out his/her willingness to participate in the construction of a substation.

In this study, a seven-point Likert-type scale, with “1” indicating “strongly disagree” and “7” denoting “strongly agree,” is used to measure six variables of willingness of the public to participate (INT), public trust (PT), attitudes to behavior (ATB), subjective norms (PNs), personal norms (SNs), and perceived behavioral control (PBC) in the substation project. Table 2 depicts the construct and items of observed variables. The standard deviation of each measured item is larger than 1, and the mean value was larger than 5.2, showing that the respondents have a higher recognition degree for the items (Qin et al., 2020). The scale design and descriptive statistics are shown in Table 3.

**Table 2:** Construct and items of observed variables.

**Sr**	**Variables**	**Number of items**	**Reference**
1	Public trust (PT)	4	Liu Y. et al., 2018
2	Attitude toward behavior (ATB)	3	Zhang et al., 2017; Rezaei et al., 2019; Qin et al., 2020
3	Subjective norms (SN)	3	Rezaei et al., 2019; Qin et al., 2020
4	Personal norms (PN)	3	Zhang et al., 2017; Qin et al., 2020
5	Perceived behavioral control (PBC)	4	Zhang et al., 2017; Rezaei et al., 2019; Qin et al., 2020
6	Willingness to participate (INT)	3	Qin et al., 2020

**Table 3:** Scale design and descriptive statistics.

**Variables**	**Item**	**Item description**	**Mean value**	**Standard deviation**
PT	PT1	I believe the site selection process of the government is fair and equitable	5.984	1.136
	PT2	I believe the existing substation equipment and technology are environment-friendly and safe	5.972	1.108
	PT3	I believe the expert analysis during decision making process of substation is reliable	5.989	1.137
	PT4	I believe the construction process will abide by relevant national laws and standards regarding environmental protection and safety	5.766	1.085
ATB	ATB1	I believe the substation construction is valuable	5.651	1.075
	ATB2	The implementation of urban substation helps improve living quality	5.724	1.064
	ATB3	Substation construction complies current social development and is worth of popularizing	5.241	1.090
SN	SN1	Supporting construction of substation helps promote social progress	5.215	1.069
	SN2	Family members or friends believe substation construction is a necessary item for correct social progress	5.419	1.128
	SN3	The power demand of current residents shall be satisfied by constructing power station	5.292	1.130
PN	PN1	Actively participating in activities planned by the government complies with my ethical principle and value belief	5.586	1.134
	PN2	I believe it is necessary to construct substation	5.588	1.141
	PN3	I will feel uncomfortable when my self-interest problems hinder urban and social development	5.548	1.228
PBC	PBC1	I believe I could participate in construction of the substation if I want to	5.813	1.385
	PBC2	I decide by myself completely whether to participate in substation construction	5.813	1.520
	PBC3	I will put forward my opinions during substation construction if I want to	5.912	1.322
	PBC4	I will insist on expressing my personal opinion on substation construction no matter hindered or not	5.842	1.328
INT	INT1	The construction of urban substation is necessary and acceptable	5.840	1.042
	INT2	If scientific site selection is conforming, I am willing to accept substation constructed nearby	5.852	1.105
	INT3	I am willing to cooperate for questionnaire before site selection, and actively make contribution	5.924	1.037

### Procedure

The structural equation model is constructed using Amos 22.0, and the data obtained from the survey are analyzed using the Decision-Making Trial and Evaluation Laboratory (Dematel) method. First, confirmatory factor analysis (CFA) is performed on the structural equation model, and then the statistical significance of the model is discussed using the reliability test and overall model fit test, and standardized path coefficients between the latent variables of the model are obtained based on the validation hypothesis. As the Dematel method is based on the assessment of correlations of influencing factors, it is a method that uses matrix tools and graph theory to effectively explore the causal relationships and systematic importance between the variables. Therefore, a direct influence matrix between the variables needs to be created, and a combined influence matrix needs to be calculated in order to analyze the logical causal relationships between the variables.

## Results

Confirmatory factor analysis (CFA) is an important part of SEM analysis. The measurement model must be tested before performing the evaluation of the structural model. A complete SEM report can only be carried out if the measurement model is reasonably acceptable. In this study, CFA is conducted for all dimensions, and the results are shown in Table 4.

**Table 4:** Reliability and convergence validity analysis.

**Variable**	**Item**	**Parameter significance estimate**				**Factor loading**	**Item reliability**	**Composite reliability**		**Convergence validity**
		**Unstd**.	**SE**	***t*** **-value**	***p*** **-Value**	**Std**.	**SMC**	**CR**	**Cronbach's α**	**AVE**
PT	PT1	1.000				0.930	0.865	0.969	0.968	0.885
	PT2	1.004	0.022	46.241	^***^	0.957	0.916			
	PT3	1.022	0.023	44.728	^***^	0.949	0.901			
	PT4	0.952	0.023	41.016	^***^	0.927	0.859			
ATB	ATB1	1.000				0.840	0.706	0.935	0.934	0.828
	ATB2	1.091	0.036	29.943	^***^	0.927	0.859			
	ATB3	1.157	0.037	31.041	^***^	0.958	0.918			
SN	SN1	1.000				0.877	0.769	0.908	0.907	0.767
	SN2	1.113	0.039	28.652	^***^	0.925	0.856			
	SN3	0.992	0.040	25.013	^***^	0.823	0.677			
PN	PN1	1.000				0.802	0.643	0.879	0.878	0.708
	PN2	1.096	0.050	21.705	^***^	0.874	0.764			
	PN3	1.144	0.054	21.378	^***^	0.847	0.717			
PBC	PBC1	1.000				0.817	0.667	0.906	0.902	0.706
	PBC2	1.053	0.050	20.958	^***^	0.784	0.615			
	PBC3	1.030	0.042	24.550	^***^	0.882	0.778			
	PBC4	1.025	0.042	24.256	^***^	0.874	0.764			
INT	INT1	1.000				0.884	0.781	0.920	0.920	0.794
	INT2	1.066	0.037	29.024	^***^	0.889	0.790			
	INT3	1.013	0.034	29.522	^***^	0.900	0.810			

*** Shows the significance under 1% levels.

### Reliability and validity test

Cronbach's α is a reliability test when using the Likert scale, and it is used to measure the consistency of respondents' score at different time periods. In this study, the reliability of questionnaire data is analyzed using SPSS24.0, and the overall Cronbach's α is 0.955, which indicates there is good internal consistency of the scale (Anderson and Gerbing, 1988). Cronbach's α of each latent variable ranges from 0.878 to 0.968, which is greater than the threshold criterion of 0.7 (Hair et al., 2017). The standardized factor loadings are all >0.7 and are significant, and the standardized factor loading squares, also known as squared multiple correlation (SMC), are all >0.5, which indicate that in the research model, the items have the high level of confidence (Casalo and Romero, 2019). Meanwhile, the construct reliability (CR) of each latent variable is >0.7 and is generally consistent with Cronbach's α (Verbeke et al., 2014). In addition, the average variance extracted (AVE) values of latent variables are all >0.7 (Fornell and Lacker, 1981), which indicate that the model has good composite reliability and convergence validity (Table 4).

**Figure 2:**
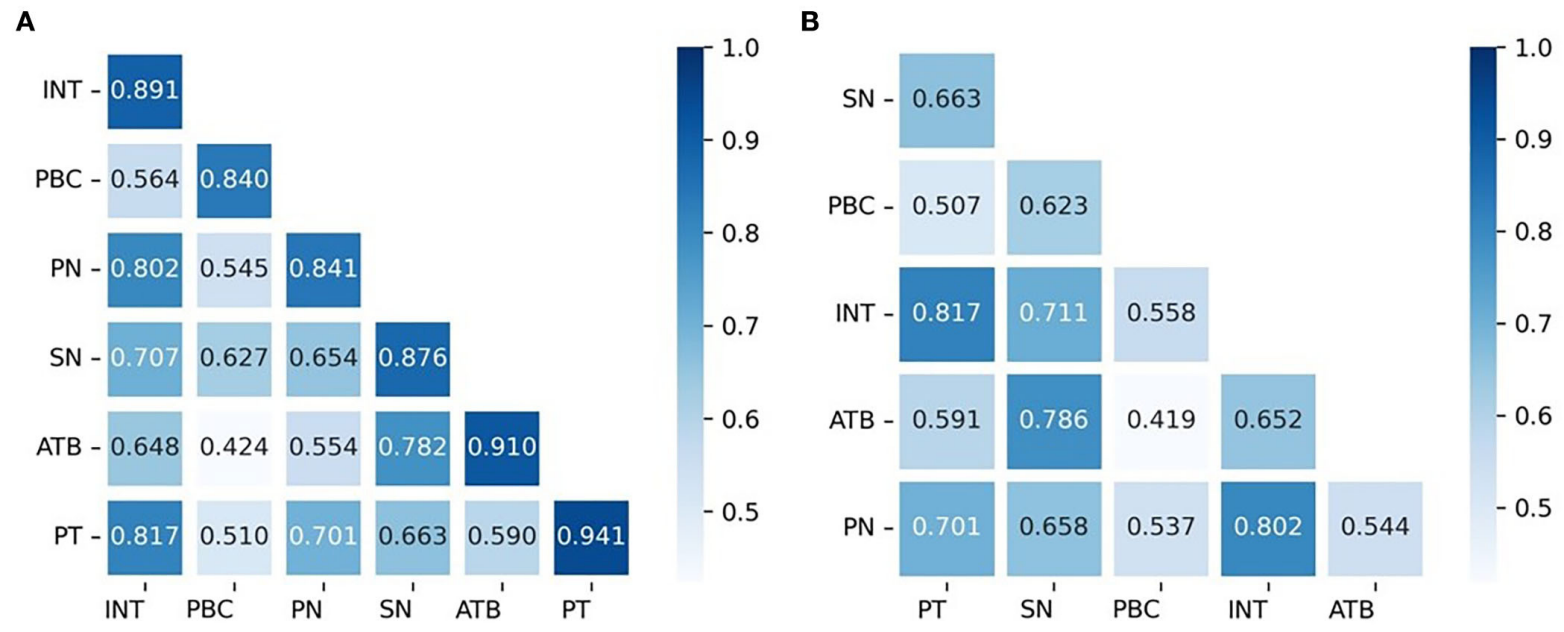
**(A,B)** Result of the discriminant validity test.

The discriminant validity analysis is performed to examine whether different two variables in the statistics are different or not, and the square roots of the AVE values on the diagonal are greater than the correlation coefficients between constructs, which means that the variables have discriminant validity (Figure 2A). In response to Henseler et al.'s (2015) suggestion that “methods that simply compare factor loadings and the square root of AVE values are flawed”, this study also use the heterotrait-to-monotrait (HTMT) ratio to evaluate the discriminant validity. The HTMT ratio is the ratio of the mean of correlations of measures between different latent variables to the mean of correlations of measures of the same latent variable. The largest HTMT value between the latent variables is 0.817 (Figure 2B), which is less than the threshold value of 0.85 (Henseler et al., 2015). Therefore, this result confirms that the variables have acceptable discriminant validity.

### Fit degree index test

**Figure 3:**
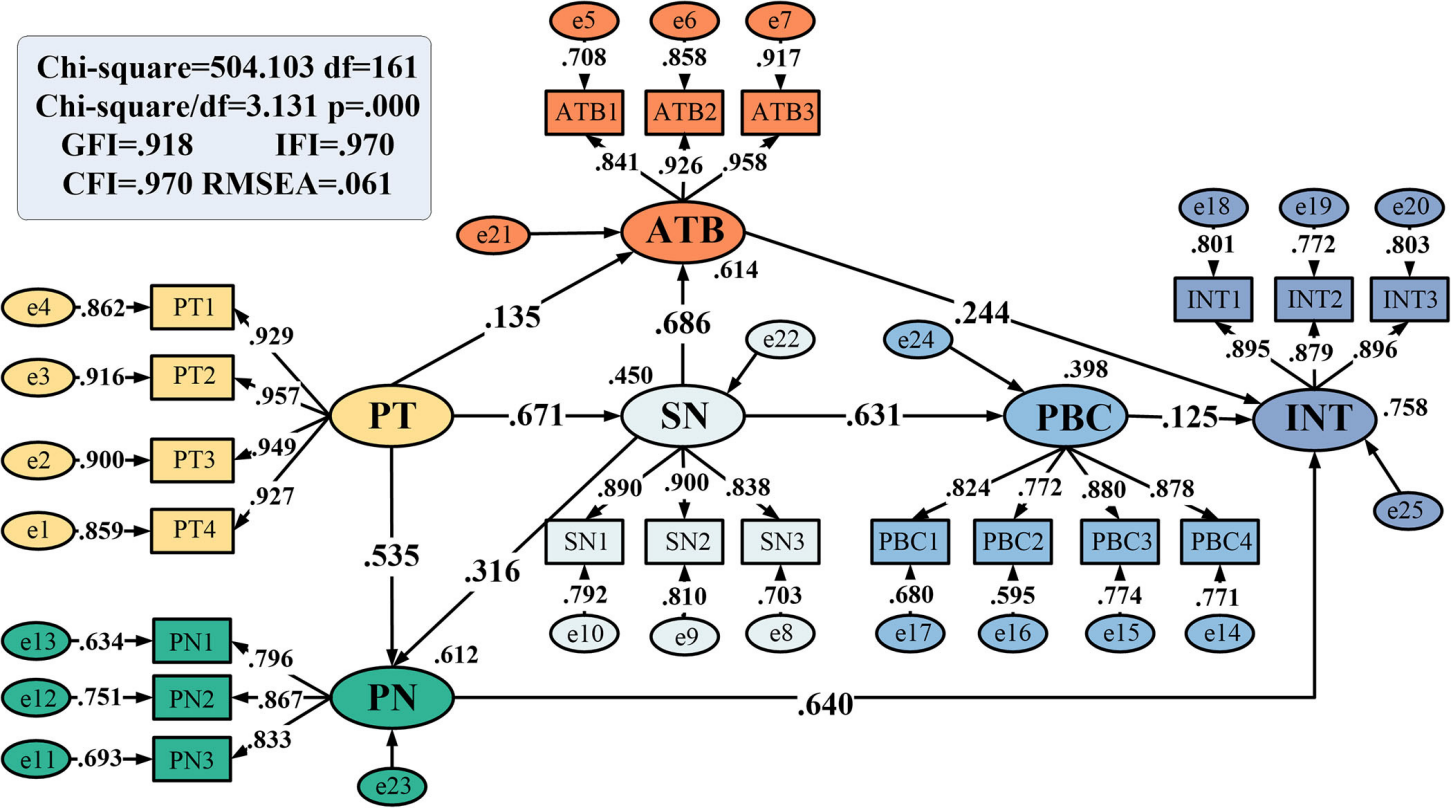
Path diagram of the integrated TPB-NAM model.

The model fit degree index refers to the model of model fitness analysis. The model path is shown in Figure 3. In the absolute fit indexes, the goodness-of-fit index (GFI) is 0.918, the root of the mean square residual (RMR) is 0.051, and the root-mean-square error of approximation (RMSEA) is 0.061, meeting all the standards of Han et al. (2010) with a GFI >0.90, and RMR and RMSEA greater than 0.08. In the incremental fit indexes, the normal fit index (NFI) is 0.957, the fitness of the comparative fit index (CFI) and the incremental fit index (IFI) are all 0.970, and all these indexes meet the standards proposed by Marsh and Hau (1996) with NFI, CFI, and IFI >0.9. In the parsimony fit indexes, the parsimony unbiased goodness-of-fit index (PGFI) is 0.704, and the parsimonious normed fit index (PNFI) is 0.811; these indexes meet the standards of Kaiser and Gutscher (2003), with the PGFI and PNFI greater than 0.5. All the fitness indicators in this study have passed (Table 5), indicating that the results of this study are acceptable.

**Table 5:** Model fit criteria and the test results.

**Index type**	**Absolute fit index**				**Incremental fit index**			**Parsimony fit index**	
	**χ^2^/*df***	**GFI**	**RMR**	**RMSEA**	**NFI**	**CFI**	**IFI**	**PGFI**	**PNFI**
Criteria	<5	>0.9	<0.08	<0.08	>0.9	>0.9	>0.9	>0.5	>0.5
Fit values	3.131	0.918	0.051	0.061	0.957	0.970	0.970	0.704	0.811
Result	Excellent				Excellent			Excellent	

### Hypothesis test

The structural path coefficient reflects the important degree of influence among dimensions; according to the model fitting results, the path coefficients for the effects of PT on ATB, SN, and PN are 0.135, 0.671, and 0.535, respectively, all of which are significantly positive, thus supporting H1, H2, and H3.

The standardized path coefficient for the SN on ATB is 0.686 and is also significant at the 1% level, and the results support H4. The SN significantly and positively influences the PN, and the standardized path coefficient is 0.316; thus, H5 is verified. Furthermore, H6 is supported as the standardized path coefficient for the PBC by the SN is 0.631. The standardized path coefficient for ATB on INT is 0.244; hence, H7 is confirmed. The standardized path coefficient of the PBC on INT is 0.125; thus, H8 is supported. In addition, among the paths that directly affect INT, the standardized path coefficient for PN is the largest, 0.640, thus supporting H9 (Table 6).

**Table 6:** Path coefficient of model.

**Hypothesis**	**Path**	**Std**.	**SE**	**C. R**.	***p*** **-Value**	**[95% conf. Interval]**	**Conclusion**
H1	PT → ATB	0.135	0.038	3.194	^***^	[0.061, 0.209]	Support
H2	PT → SN	0.671	0.038	16.779	^***^	[0.597, 0.745]	Support
H3	PT → PN	0.535	0.047	11.460	^***^	[0.443, 0.627]	Support
H4	SN → ATB	0.686	0.048	13.717	^***^	[0.218, 0.414]	Support
H5	SN → PN	0.316	0.050	6.799	^***^	[0.527, 0.735]	Support
H6	SN → PBC	0.631	0.053	14.718	^***^	[0.170, 0.318]	Support
H7	ATB → INT	0.244	0.038	6.679	^***^	[0.170, 0.318]	Support
H8	PBC → INT	0.125	0.026	3.809	^***^	[0.074, 0.176]	Support
H9	PN → INT	0.640	0.038	15.381	^***^	[0.566, 0.714]	Support

*** Shows the significance under 1% levels.

### Dematel causality test

**Figure 4:**
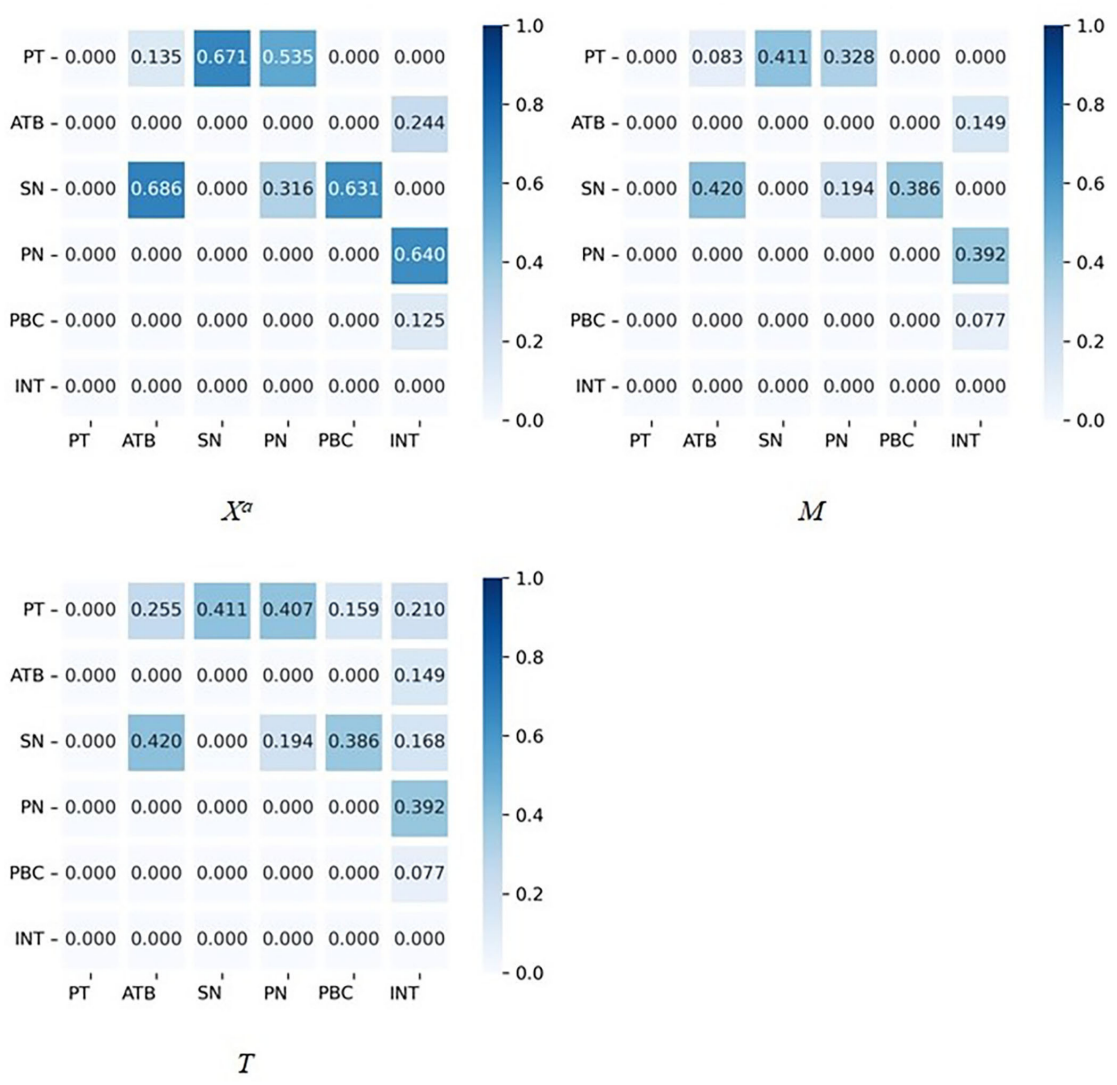
Calculation results of direct influence matrix, normalized matrix, and comprehensive influence matrix. ***X***^***a***^**:** Calculation results of direct influence matrix. ***M***: Calculation results of normalized matrix. ***T***: Calculation results of comprehensive influence matrix.

On the basis of the obtained standardized path coefficients for each latent variable of the SEM, the logical relationships and path influence degree among the latent variables are further analyzed by the Dematel method. Specific steps are as follows, and the calculation results are depicted as Figure 4.

Step 1: The direct influence matrix *X*^*a*^ is calculated. The direct influence matrix *X*^*a*^ can be obtained by quantifying the influence relationship between the variables in the model. The elements in *X*^*a*^ are the path coefficients between the variables, and a larger value of an element indicates a greater degree of influence between variables.
Step 2: The normalized matrix *M* is calculated. The elements of each row in *X*^*a*^ are summed, and the maximum value of which is chosen as *Max* (*n*); moreover, the elements in *X*^*a*^ divided by *Max*(*n*), and the normalized matrix *M* can be obtained.
Step 3: The comprehensive influence matrix *T* is calculated. The normalized matrix *M* is introduced into the formula *T*= *M* (1 – *M*)^−1^ to obtain comprehensive influence matrix *T*. Furthermore, the sum of the rows of *T* matrix represents the degree of influence of each variable, and the sum of the columns of the *T* matrix represents the degree to which each variable is influenced.

**Figure 5:**
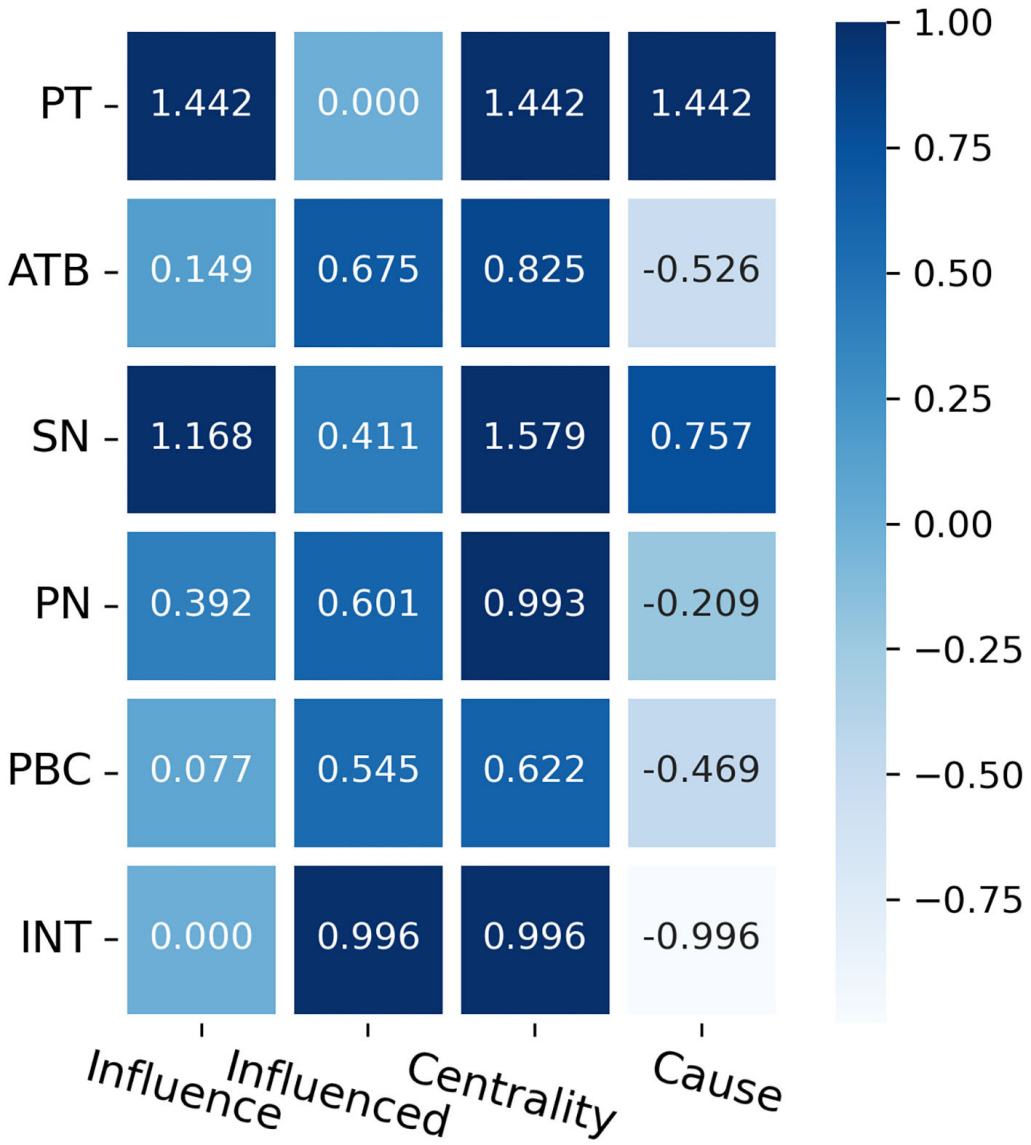
Influence degree, influenced degree, centrality, and cause degree.

The centrality degree is the magnitude of the variable's role in the system, this indicator is expressed by the sum of influence degree and influenced degree, and the cause degree is the difference between the influence degree of the variable and the influenced degree. The centrality degree of the SN is the largest, followed by PT, INT, PN, and ATB (Figure 5).

## Discussion

Previous research (Mancha and Yoder, 2015; Ho and Oshita, 2019) found more emphasis on egoism and SN and neglect of altruism and PN when discussing the NIMBY effect, and the current work covers this gap. It demonstrates that the integrated TPB-NAM model exhibits a better model fit, which means that the model has improved applicability for studying the behavior and willingness of the public to participate in NIMBY projects such as substations, as well as providing a more comprehensive analysis of the influencing factors and mechanisms.

The present study further expands on the research paradigm that Qin et al. (2020) developed by including PT as an antecedent variable. Because there are knowledge differences between the public and experts regarding NIMBY projects such as substations, the decision-making of the public is related to their trust in the local government and experts. After causality testing, the findings show that PN is the variable with the largest causality degree, and it has a relatively large influence degree and centrality degree. In brief, the results of this empirical study verify that the PN is the core variable in the model, and its enhancement positively affects the other dimensions, ultimately contributing to improvements in public willingness to participate.

The SN does not have a direct effect on public willingness to participate in substation projects but indirectly acts through ATB, PN, and PBC. The results of the empirical study show that the good activation effect of the SN on ATB, PN, and PBC is verified, which is consistent with the results of other studies on public participation in public infrastructure projects (Zhang et al., 2017). According to the findings of this study, when the public is involved in the construction of a substation, the PN is a direct determinant influencing one's willingness to participate, and the path coefficient is the largest. The empirical findings are consistent with the results of Rezaei et al.'s (2019) study that PN as the basic variable of moral responsibility will stimulate altruism of the public and thus influence their willingness to participate. It is worth noting that regarding the value of the cause degree, the results of PT and SN are positive, which are cause elements and active influence factors, while the results of ATB, PBC, and INT are negative, which are outcome elements and influenced factors.

## Conclusion

To develop and enrich existing research fields, based on the planned behavior theory and normative activation theory, this research constructs a new theoretical model, that is, the integrated TPB-NAM model, which uses the structural equation model to explore factors influencing willingness of the public to participate in substations and logic relationships between the variables, and introduces public trust to explain the specific impact mechanism, which is an antecedent variable of attitudes, subjective norms, and personal norms. At the same time, we pay attention to the role that subjective norms play in this study; it has a positive impact on the activation of attitudes and personal norms. The results of this study have theoretical and practical implications for understanding the determinants that influence public participation in substations and contribute to reducing the conflicts between the public and the government due to the NIMBY effect.

### Theoretical contributions

Compared with the existing literature, this article is innovative in the following aspects:

First, previous articles on the NIMBY effect have mainly focused on the theory of planned behavior or normative activation theory (Stewart and Aitken, 2015; Neukirch, 2016), with less focus on integrating the two theories. This study develops an integrated TPB-NAM model and demonstrates the model fit and applicability. This study not only enriches the theoretical background in behavioral research but also provides a valid research model for analyzing the behavior and willingness of the public to participate in NIMBY projects such as substations and the specific issues that arise from this.
Second, this study improves the research framework originally designed by Rezaei et al. (2019) by adding personal norms variables, taking into account behavioral attributes including egoism and altruism. Also, this study modifies the research idea originally designed by Qin et al. (2020) by adding public trust as an antecedent variable to make the theoretical model more predictive and explanatory.
Third, in order to overcome the shortcomings of previous studies limited to analyzing the influence factors at the personal level (Schumacher and Schultmann, 2017; Johnson et al., 2018; Martínez-Mendoza et al., 2021), this study explores and tests the interaction at the level of elements such as personal norms, public trust, and subjective norms, as well as their influence on public behavior and willingness. This research breaks the gap of previous related research perspectives and provides new ideas for discussing the driver factors that influence the implementation of substation projects.

### Practical implications

This research provides a valuable reference for resolving conflicts arising from NIMBY projects such as substations in China, as described in the following text.

First, in the process of building a substation, if there is inadequate disclosure of information such as the rationality and hazards of the location, this will exacerbate the negative sentiment of the public and lead them to irrational behavior. Therefore, it is essential to establish a public trust mechanism to reduce the conflict caused by the NIMBY effect during the construction of substations. The government and other relevant departments need to respond to public demands in time to strengthen the trust relationship and weaken the NIMBY effect by disclosing the risks in detail and taking corresponding social responsibilities.

Second, it is important to change the identity of the public from an altruistic perspective to motivate public participation in substation projects. The substation is a project with the NIMBY effect, and the potential risks it may bring can disrupt the benefit–cost balance between society and individuals. Therefore, the government needs to strengthen the cultivation of public moral responsibility and, at the same time, provide more opportunities for the public to participate in the construction of NIMBY projects, highlight their social identity, and activate the public sense of concession of interest.

Third, conflicts arising from NIMBY projects are concentrated outbursts of abnormal emotions after the gathering of individuals. The early understanding of the public of the event will be infected by the emotions of those around them, and a few unstable individuals will drive the direction of collective emotions, forming large-scale irrational conflicts. Therefore, considering the different educational backgrounds of the public, the government should increase its efforts to publicize the substation project and eliminate the negative externalities caused by wrong public opinions and knowledge structure through publicity so that the public can form a good social consensus on the NIMBY projects and further create a social atmosphere to support the substation project, which will help the substation project move forward steadily.

### Limitations and future recommendation

As with any other research, the findings of our study should be interpreted with certain limitations in mind. First, willingness and behavior of the public in participating in the construction of substations is influenced by the complex interaction of many social and economic factors, such as one's endogenous factors and the external environment. The integrated TPB-NAM model proposed in this article is innovative, but it is constrained by objective conditions and may omit some explanatory variables. In future research, other explanatory variables can be explored to improve the explanatory validity of the theoretical model in the context of realistic NIMBY problems.

Second, the sample used in this study is obtained exclusively from China, but there are differences in economic development and educational environments in different countries and regions. Whether these differences affect willingness of the public to participate in substations projects needs to be further studied. The comparative analysis of the data obtained from different countries or regions can be performed to further validate and improve the model.

Third, the following questions remain to be solved in future studies: are there significant differences in public trust in terms of gender, age, and literacy level? Are there significant group differences in participation willingness with respect to one's characteristics? Thus, the heterogeneity of one's willingness to participate in the substations projects needs to be further discussed.

## Data availability statement

The original contributions presented in the study are included in the article/supplementary materials, further inquiries can be directed to the corresponding author.

## Ethics statement

The studies involving human participants were reviewed and approved by the Research Ethics Committee of the North China University of Water Resources and Electric Power. The patients/participants provided their written informed consent to participate in this study.

## Author contributions

XM and JL: conceptualization, formal analysis, writing—original draft preparation, and writing—review and editing. XM: methodology and supervision. JL: software. XM, JL, and TC: validation. FX and WW: resources. CC: data curation. XM and FG: funding acquisition. All authors have read and agreed to the published version of the manuscript.

## Funding

This research was partially funded by Zhengzhou Science and Technology Collaborative Innovation Special Project. This research was also funded by the Key Soft Science Projects in Henan Province (Grant No.222400410010), A Research Project Study on the Forecast and Analysis of Carbon Emissions in the Construction Sector in Henan Province (No. 222400410010).

## Conflict of interest

The authors declare that the research was conducted in the absence of any commercial or financial relationships that could be construed as a potential conflict of interest.

## Publisher's note

All claims expressed in this article are solely those of the authors and do not necessarily represent those of their affiliated organizations, or those of the publisher, the editors and the reviewers. Any product that may be evaluated in this article, or claim that may be made by its manufacturer, is not guaranteed or endorsed by the publisher.
